# Androgen receptor signaling regulates follicular growth and steroidogenesis in interaction with gonadotropins in the ovary during mini-puberty in mice

**DOI:** 10.3389/fendo.2023.1130681

**Published:** 2023-04-19

**Authors:** Marie M. Devillers, Charlotte M. François, Mélanie Chester, Raphaël Corre, Victoria Cluzet, Frank Giton, Joëlle Cohen-Tannoudji, Céline J. Guigon

**Affiliations:** ^1^ Université Paris-Cité, CNRS, Inserm, Biologie Fonctionnelle et Adaptative, Paris, France; ^2^ AP-HP, Pôle biologie-Pathologie Henri Mondor, Inserm IMRB U955, Créteil, France

**Keywords:** mini-puberty, ovary, androgens, FSH receptor, estradiol, follicular growth

## Abstract

In females, androgens contribute to ovarian diseases such as polycystic ovarian syndrome (PCOS), but their action is also crucial for ovarian physiology, i.e., follicular growth and estradiol (E2) synthesis during reproductive life, in interaction with the gonadotropins LH and FSH. However, it is unclear whether androgens already play a role in the ovary at mini-puberty, a phase of postnatal development with active follicular growth and high E2 levels. Therefore, we analyzed the potential actions of androgens on the ovary and their possible interaction with gonadotropins during this period in mice. We used molecular-based studies and pharmacological approaches *in vivo* and on cultured ovaries. We found that mini-pubertal ovaries produce significant amounts of testosterone and display androgen receptor (AR) expression in growing follicles, both under the control of LH. By blocking AR signaling either *in vivo* or in ovarian cultures, we found that this pathway may participate in the regulation of prepubertal E2 synthesis and follicular growth, possibly by regulating the expression of a number of key intra-ovarian regulators, including FSH receptor (*Fshr*), the aromatase enzyme converting androgens into estrogens (*Cyp19a1)* and the cell cycle inhibitor p27KIP1 (*Cdkn1b)*. We further showed that AR may stimulate FSH-mediated regulation of *Cyp19a1* through its action on *Fshr* mRNA abundance. Overall, this work supports the idea that AR signaling is already activated in mini-pubertal ovaries to regulate E2 synthesis and follicular growth, at the interplay with LH and FSH signaling. Its early action may, thus, contribute to the implementation of early ovarian function with possible impacts on reproductive function.

## Introduction

Shortly after birth in female mammals, the levels of the gonadotropins luteinizing hormone (LH) and follicle-stimulating hormone (FSH) transiently increase together with significant ovarian activity, as demonstrated by the sustained production of estradiol (E2) and the presence of numerous growing follicles (1–5). Because this peculiar hormonal profile resembles that observed at puberty, this developmental period has been named “mini-puberty of infancy” (6, 7). Excessive mini-puberty can be observed in girls, essentially preterm babies, who show even higher surges of gonadotropins, estradiol, dehydroepiandrosterone (DHEA) and testosterone than babies born at full-term (8–10). This altered hormonal profile can be associated with aberrant mammary gland growth, appearance of pubic and axillary hair and vaginal bleeding (11).

Although the physiological role of mini-puberty remains elusive, a number of evidence suggest that this phase plays a role in reproductive function by acting postnatally through E2 signaling in the hypothalamus, the uterus, and the mammary gland (12). How E2 synthesis is regulated during mini-puberty is, therefore, a key issue to understand the developmental events involved in programming reproductive function, and the etiology of reproductive disorders linked to alterations of ovarian activity at this stage. There are, however, very few studies aiming at understanding the regulation of the ovary before reproductive life.

Our previous studies have demonstrated in the mouse that the high circulating FSH levels of mini-puberty are essential to ovarian steroidogenesis by inducing the expression of the cytochrome P450 aromatase (*Cyp19a1*) in the population of follicles located in the center of the ovary to promote E2 synthesis (4). These follicles, which began to develop immediately after their formation at birth, belong to the so-called “first follicular waves” reaching the preantral/early antral stage at mini-puberty and appear to be highly FSH-responsive unlike the other growing follicles located at the periphery (4, 13). They contribute to the first ovulations at puberty and at the beginning of reproductive life (14, 15). We previously found that at the high concentrations of mini-puberty, FSH is unable to promote granulosa cell proliferation in preantral/early antral follicles, indicating that other mechanism(s) are involved in this process (4). In the present study, we investigated the possible role played by androgens, which are known regulators of ovarian function during reproductive life (16). Indeed, *in vivo* and *in vitro* studies suggest that androgens promote E2 synthesis and up-regulates the transcription of FSH receptor gene (*Fshr*) in the adult ovary (17–19). In addition, androgens regulate follicular growth in the adult ovary, notably by stimulating the transition from the preantral to the antral stage (20–22). Androgens exert these aforementioned actions, not necessarily after conversion to estrogens, but through their binding to the nuclear androgen receptor (AR) (20–22). In ovaries from prepubertal and adult cycling females, testosterone production is regulated by LH, which acts on thecal cells to enhance the expression of components of androgen biosynthesis pathway (23–25). Taken together, these observations led us to test the hypothesis that androgens regulate both ovarian steroidogenesis and follicular growth during mini-puberty by acting through AR, possibly in interplay with FSH and LH.

To address this question, we analyzed the intra-ovarian content and circulating levels of testosterone as well as AR expression in the prepubertal mouse, and their regulation by LH. We investigated the effects of androgens on FSH-mediated action on follicular growth and steroidogenesis in the ovary at mini-puberty by manipulating AR signaling through pharmacological approaches with an AR antagonist both *in vivo* and in ovarian cultures.

## Materials and methods

### Animals and treatments

Studies were conducted on C57BL/6JRj mice aged 7 to 27 days post-natal (dpn) and on adult females that were born at the animal facility from genitors purchased at Janvier Labs (Le Genest St Isle, France) (n=180 mice in total). The day of birth was designed as 0 dpn. Mice were maintained under controlled conditions (12h light/dark cycle) with food (Scientific Animal Food and Engineering (SAFE), A03-10) and water available *ad libitum*. Ten µg of GnRH antagonist (Ganirelix, Orgalutran^®^, N.V. Organon, Puteaux, France) or saline were subcutaneously injected twice on prepubertal female mice at 12 and 13 dpn, as previously described (4). In addition to Ganirelix, 13 dpn mice received an intraperitoneal injection of 5 U.I. human chorionic gonadotropin (hCG) (N.V.Organon), and were killed at 14 dpn. Injections were performed with Hamilton syringes connected with catheters and needles (Phymep, Paris, France). For another study, mice were subcutaneously injected with an androgen receptor antagonist, flutamide (5 mg/kg; F9397, Sigma), dissolved in corn oil (Sigma) every 10 hours starting at 12 dpn until the day of dissection (14 dpn) (total: 4 injections). Adult females were killed on the day of diestrus 1 or 2 determined by vaginal smears, as described (4). Mice were anesthetized with a mix of ketamine (Imalgene^®^ 1000) and xylazine (Rompun^®^ 2%) to collect the blood by cardiac puncture. After cervical dislocation, ovaries were either frozen in liquid nitrogen and stored at -80°C for RNA extraction, fixed in 4% paraformaldehyde (PFA) for immunofluorescence studies or immediately used for organotypic cultures. Blood was allowed to clot at room temperature for at least 15 minutes, and then centrifuged at 4°C, 5000 g for 5 minutes to obtain serum. Experiments were performed in accordance with standard ethics guidelines and were approved by Institutional Animal care and Use committee of the University Paris Cité and by the French Ministry of Agriculture (agreement #04015.01).

### RNA extraction, reverse transcription, and quantitative real-time PCR

Single frozen ovaries were lysed with TissueLyser II (Qiagen, Courtaboeuf, France) in RLT buffer from RNeasy mini kit (#74106, Qiagen) containing 1% β-mercaptoethanol, following manufacturer’s protocol. Total RNA extraction was performed on columns and eluted with 30 µl of sterile water. Total RNA (120 to 250 ng) was reverse-transcribed with Superscript II (Invitrogen, Cergy Pontoise, France) and Random Primers (Invitrogen) following manufacturer’s instructions. Primers used for quantitative real-time PCR are listed in **Table 1**. *Ppid* (Cyclophilin D) was used as a reference gene to normalize the data. Real-time PCR was performed with Lightcycler 480 SYBR Green I Master and LightCycler instrument (Roche Molecular Biochemicals, La Rochelle, France), following the MIQE guideline [https://pubmed.ncbi.nlm.nih.gov/19246619/]. Experiments were performed with at least four different ovarian samples/age or treatment group, with each sample run in triplicate as previously described (26).

**Table 1:** Primers used for quantitative RT-PCR analysis.

Gene	Forward primer	Reverse primer
*Ar*	GCTCACCAAGCTCCTGGATT	TCAGGAAAGTCCACGCTCAC
*Star*	GTCATCAGAGCTGAACACGG	TGGCGAACTCTATCTGGGTC
*Cyp11a1*	GCACTTTGGAGTCAGTTTACATC	AGGACGATTCGGTCTTTCTT
*Cyp17a1*	CTTTCCTGGTGCACAATCCT	TACGCAGCACTTCTCGGATA
*Hsd3b1*	CAGCCAGGGGCCTTCGAGAC	GCTGGCATTAGGGCGGAGCC
*Ppid*	CAAAGTTCCAAAGACAGC	CTGGCACATGAATCCTGGAA
*Ccnd1*	GAFATTGTGCCATCCATG	CTCCTCTTCGCACTTCTGCT
*Ccnd2*	CAGAAGGACATCCAGCCGTAC	TCGGGACTCCAGCCAAGAA
*Cdkn1b*	GTTAGCGGAGCAGTGTCCA	TCTGTTCTGTTGGCCCTTTT
*Cyp19a1*	TACTTCATGTTACTTCTCGTCGC	TATCCTCGATCTTTATGTCTCTGTCAC
*Fshr*	CTGGCATTCTTGGGCTCG	GGGCGGAATCTCGGTCA
*Amh*	TGCTAGTCCTACATCTGGCTGA	GTCCAGGGTATAGCACTAACAGG

Ar, androgens receptor; Star, steroid acute regulatory protein; Cyp11a1, cytochrome P450 family 11 subfamily A member 1; Cyp17a1, cytochrome P450 family 17 subfamily A member 1; Hsd3b1, hydroxy-delta-5-steroid dehydrogenase, 3 beta-and steroid delta-isomerase 1; Ppid, peptidylprolyl isomerase D/cyclophilin D; Ccnd1, cyclin D1; Ccnd2, cyclin D2; Cdkn1b, cyclin dependent kinase inhibitor 1B/p27KIP1; Cyp19a1, cytochrome P450 family 19 subfamily A member 1/aromatase; Amh, anti-müllerian hormone; Fshr, follicle stimulating hormone receptor.

### Culture of postnatal ovaries

Ovaries from 13 dpn female mice were placed on cell culture inserts (Millicell #PICM01250, Millipore, Guyancourt, France) on the top of 400 µl of RPMI culture medium without red phenol containing fetuin (Sigma, #F2379), insulin (Sigma, #I0516), transferrin (Sigma, #T8158), sodium selenite (Sigma, #S5261) and BSA (Euromedex, Souffelweyersheim, France) in 24-well plates for 1 hour. Then, the culture medium was replaced with 400 µl of fresh medium containing either or in combination: recombinant FSH (500 ng/ml, Gonal-F, Merck Serono), hydroxyflutamide (10 µM; H4166, Sigma), forskolin (10 µM, F39147, Sigma). After 8 hours of treatment, ovaries were snap-frozen in liquid nitrogen and stored at -80°C for RNA extraction.

### Determination of testosterone levels

The mass spectrometry coupled with gas chromatography (GC-MS) procedure to determine testosterone content in the serum and in the ovary was performed as described in previous papers (4, 26). Briefly, frozen mouse ovaries were introduced in a Lysing Matrix D tube (MP Biomedicals, Illkirch-Graffenstaden, France) with 0.5 ml of cold saline water and 10 μl deuterated methanol internal standard working solution. The sample was homogenized with an FP120-HY-230 Ribolyser (Hybaid, Teddington, Middlesex, UK) for three times of 20 seconds at maximum speed. After centrifugation, the liquid phase with the ceramic spheres was collected in a clean glass tube. The tissue residuals in Lysing Matrix tube were recovered with 3 ml of 1-chlorobutane (HPLC grade). After fast centrifugation, the upper organic phase was collected on conditioned Hypersep SI 500 mg SPE minicolumn (Thermo Scientific, Rockwood, USA). The column and adsorbed material were then washed with ethyl acetate/hexane (6 ml; 1/9, v/v). The second fraction containing testosterone was eluted using ethyl acetate/hexane (4 ml; 1/1, v/v), then evaporated at 60°C to dryness. Testosterone was derivatized with pentafluorobenzoyl chloride (PFBC) (103772-1G, Sigma-Aldrich, Steinheim, Germany). Final extracts were reconstituted in isooctane, then transferred into conical vials for injection into the GC system. A quadrupole mass spectrometer equipped with a chemical ionization source (NCI) and operating in single ion monitoring mode (SIM), was used for detection (HP5973, Agilent Technologies, Massy, France). The linearity of steroid measurement was confirmed by plotting the ratio of the steroid peak response/internal standard peak response to the concentration of steroid for each calibration standard. Lower limit of quantification was 1.15 pg. The intra- and inter-assay variability of the low limit of quantification CVs was 13.3 – 20%.

### Gonadotropin measurements

FSH and LH were simultaneously assayed in 10 µl of serum simplicate of the same serum samples using the Luminex technology with the mouse pituitary magnetic bead panel Milliplex MAP kit (Merck-Milipore, Nottingham, UK) in accordance with the manufacturer’s instructions. Samples were run on a Bioplex-200 instrument (Bio-Rad, Marne-La-Coquette, France) and concentrations were calculated using a-five parameters logistic fit curve (5PL) generated from the standards by the Bio-Plex Manager 6.1 software (Bio-Rad, Marne-La-Coquette, France). The sensitivity of the assays was 32 pg/ml of FSH and 3.2 pg/ml for LH. The inter-assay coefficient of variation was 5.4% for FSH and 3.2% for LH. The intra-assay coefficient of variation was 7.6% for FSH and 5.9% for LH.

### Tissue processing for histological analyses

For histological analyses, at least five ovaries of 14 dpn mice treated with either flutamide or its vehicle were fixed for 1-2 hours in Bouin’s solution, rinsed in PBS and placed in ethanol 70%. The ovaries were then dehydrated using increasing concentrations of ethanol (70%-95%-100%) and paraffin-embedded using standard protocols. Sections of 5-µm thickness were mounted on glass slides at the frequency of one every three sections, and they were stained with hematoxylin/eosin (HE), using routine procedures. After dehydration in alcohol and mounting in Eukitt (Sigma), slides were scanned using Axio Scan Z1 Zeiss at Pasteur Institute (Histology platform, Johan Bedel). Follicle classification was the same as that described in (14), with some modifications. Briefly, primordial follicles were constituted by a resting oocyte surrounded by a single layer of flattened granulosa cells. Primary follicles contained a growing oocyte with a single layer of cuboidal or mixed flattened/cuboidal granulosa cells. Preantral follicles contained an oocyte surrounded by at least two layers of granulosa cells, and no antrum or spaces between cells. Follicles were classified as early antral when they contained a growing oocyte enclosed by at least two layers of granulosa cells with either scattered areas of follicular fluid or a single antral cavity. We also monitored follicular atresia in preantral and early antral follicles, and searched for oocyte fragmentation, disordered granulosa layers, pycnotic granulosa cells (at least 3/follicle) and hypertrophied theca layer. Follicular counting was performed on every three tissue sections. To avoid repeated counting of the same preantral/antral follicle on several tissue sections, when a follicle was seen for the first time, it was marked and tracked in each subsequent section throughout which it appeared. Only follicles with visible oocytes were counted. Follicle sizes were determined by the measurement of their areas on HE-stained ovarian tissue sections using Orbit image analysis software. Follicles with oocytes exhibiting a visible nucleus were manually outlined and cross-sectional area measurement was calculated by Orbit software, from 6 representative slides for each ovary.

### Tissue processing for immunofluorescence

Mouse control ovaries were rapidly collected and fixed for 1 hour in 4% paraformaldehyde (PFA), rinsed in PBS and placed in sucrose 18% and then in a drop of tissue-Tek O.C.T. compound (Sakura) for preparing frozen sections, as described previously (14). Sections of 7-μm thickness were mounted on glass slides. For each antibody tested, all ovarian sections were stained in one run to compare the immunofluorescence staining among the different studied groups. Frozen sections were rehydrated in PBS and incubated for one hour with 10% (wt/v) normal goat serum in PBS. Following removal of the goat serum, sections were incubated overnight at 4°C in a humid chamber with anti-rabbit polyclonal primary antibodies diluted in PBS-BSA 0.5% (**Table 2**). They were rinsed three times in PBS at RT, and then incubated for one hour at RT in goat anti-rabbit IgG (H+L) secondary antibodies (ref# A21069, lot #2146040, Invitrogen, dilution 1/1000 in PBS-BSA 0.5%). Control sections were incubated with isotype IgG (Vector Laboratories), instead of the respective primary antibodies, according to the manufacturer’s instructions. Ovarian sections were then rinsed in PBS and mounted in an anti-fading medium containing DAPI (Sigma) for observation with a Nikon Eclipse 90i. The images were processed with ImageJ software to establish the % of Ki-67 positive granulosa cells per follicle (from 8 preantral/antral follicles in 4 control and 4 flutamide-treated mice). This was obtained by normalizing the number of Ki-67 positive granulosa cells by the total number of granulosa (DAPI+) cells. Granulosa cell counting was performed using the “Cell Counter” plugin of the software.

**Table 2:** List of the antibodies used for immunofluorescence.

Name	Species	Reference	Dilution	Lot #
Anti-androgen receptor	Rabbit monoclonal	ab133273	1/300	GR3271456-7
Anti-cleaved caspase-3 (Asp 175) (5A1E)	Rabbit monoclonal	9664T (Apoptosis Antibody Sampler Kit #9930T)	1/1000	21
Anti-Cyclin D2 (M-20)	Rabbit polyclonal	sc-593	1/200	A2716
Anti-AMH	Rabbit monoclonal	ab272221	1/100	GR3337320-1
Anti-p62/SQSTM1	Rabbit monoclonal	ab109012	1/400	GR3425465-11
Anti-ATG-7	Rabbit monoclonal [EPR6251]	ab133528	1/400	GR3421998-4
Anti-Ki-67	Rabbit polyclonal	ab15580	1/1000	GR153088-1

### Statistical analyses

For each experiment, the number of samples per age/treatment group is indicated in the legend of the figure. Data were analyzed using Prism 6 (version 6.0, GraphPad Software). Depending on experimental setting, statistical analyses were performed by Student t-test (one parameter and two groups), one-way ANOVA (one parameter and more than two groups) when data showed normal distribution as evaluated by the Shapiro-Wilk test, or non-parametric Mann-Withney (two groups) or Kruskal-Wallis test (more than two groups). For analyses of at least two parameters, two-way ANOVA was used. Data are shown as means ± SEM. A *P* value < 0.05 was considered as significant.

## Results

### Ontogenesis of testosterone production and AR expression in the ovary before puberty

**Figure 1:**
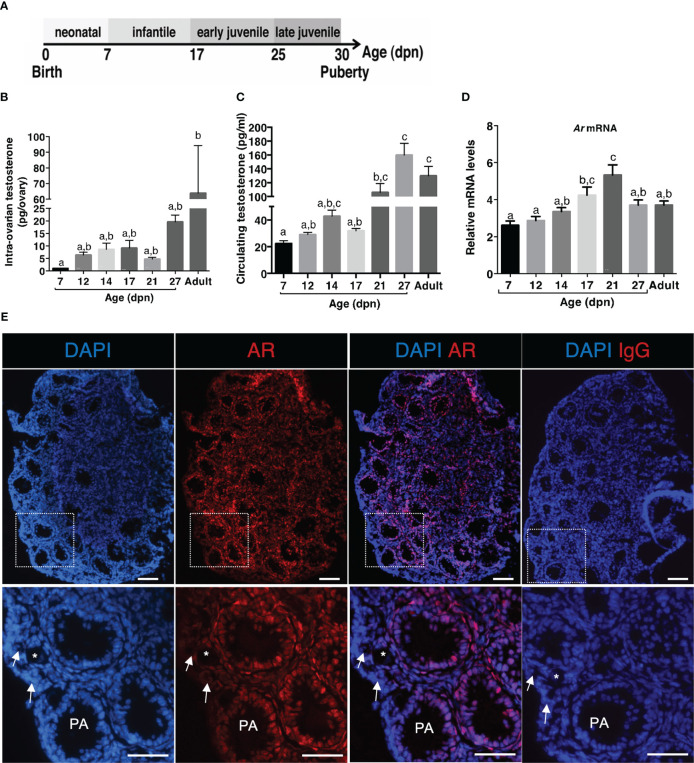
Ontogeny of androgen production and responsiveness in the ovary during the prepubertal period **(A)** Schematic representation of the different periods occurring in mice before puberty. **(B)** Intra-ovarian contents of testosterone were measured by GC-MS during the infantile (7-17 dpn), the early juvenile (21 dpn) and the late juvenile periods (27 dpn) and in adult females (4 to 6 ovaries from different females/group). **(C)** Serum testosterone levels were measured by GC-MS during the infantile (7-17 dpn), the early juvenile (21 dpn) and the late juvenile periods (27 dpn) and in adult females from 6 different samples at each age (7-21 dpn: pooled serum from 3-4 females, 27 dpn and adults: individual samples). **(D)** Relative intra-ovarian abundance of *Ar* transcripts in infantile (7-17 dpn), early juvenile (21 dpn) and late juvenile (27 dpn) and in adult females was determined by quantitative real-time RT-PCR and normalized to the mRNA levels of the housekeeping gene *Ppid* (Cyclophilin D) (6 ovaries/group). **(E)** Analyses of AR distribution in a 14 dpn mouse ovary by immunofluorescence with anti-AR antibodies (positive cells appear as red) and nuclear staining with DAPI (blue fluorescence). Merged images were obtained by ImageJ. The bottom panel is a magnification of the rectangle areas shown on the top. Preantral and early antral follicles show a significant AR nuclear staining in granulosa and thecal cells. Primary follicles (*) exhibit AR-positive granulosa cells, while primordial follicles (white arrows) are devoid of stained cells. Bars: 100 µm. In graphs, bars are the means ± SEM. Data were analyzed using a one-way non-parametric ANOVA test (Kruskal-Wallis in B and C, Tukey’s Multi Comparison test in D). Distinct letters indicate significant differences between ages.

We specified prepubertal levels of testosterone in ovaries and serum, by selecting females at different periods before puberty: 7 dpn (neonatal), 12 and 14 dpn (infantile), 17 and 21 dpn (early juvenile) and 28 dpn (late juvenile) (**Figure 1A**). We used the GC-MS technology which is one of the most reliable methods to measure sex steroids. The results showed that intra-ovarian testosterone is produced as early as the neonatal period, reaching approximately 1 to 9 pg/ovary between 7 dpn and 21 dpn. It was lower at these developmental stages than in juvenile and adult females, reaching about 30 pg/ovary and 62 pg/ovary, respectively (**Figure 1B**). Testosterone was detected in the serum as early as 7 dpn (about 22 pg/ml), and it increased to about 48 pg/ml at 14 dpn (**Figure 1C**). Circulating testosterone levels further increased in late juvenile and adult females, reaching about 125-150 pg/ml (**Figure 1C**).

We then studied the expression of AR and by using RT-qPCR, we detected *Ar* transcripts throughout the prepubertal period (**Figure 1D**). We observed that *Ar* expression progressively increased from 7 to 21 dpn (**Figure 1D**). To specify AR protein expression pattern in the mouse ovary at mini-puberty, we performed immunofluorescence studies with a specific anti-AR antibody. We found AR expression in the nucleus of granulosa cells of follicles from the primary stage onwards and in thecal cells, but not in oocytes (**Figure 1E**). Variable levels of AR staining were observed in granulosa cells, suggesting different androgen receptivity between cells. No AR staining was observed in the population of primordial follicles (**Figure 1E**).

### Gonadotropin-mediated regulation of testosterone synthesis and AR expression during mini-puberty

**Figure 2:**
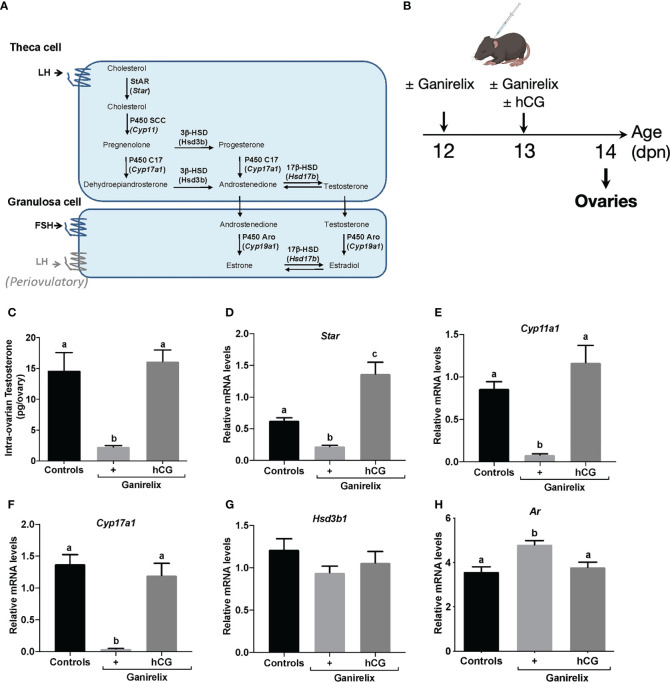
Impact of the high LH levels of mini-puberty on testosterone production and androgen receptivity in the ovary **(A)** Schematic representation of ovarian steroidogenesis in thecal and granulosa cells. **(B)** Experimental design for Ganirelix treatment used to decrease serum gonadotropins and replacement of LH by hCG injection. **(C)** Intra-ovarian contents of testosterone were measured by GC-MS in ovaries of infantile females treated with saline solution (controls), Ganirelix (+) or Ganirelix + hCG (+/hCG) (6 to 8 ovaries from different females/group). **(D–H)** Relative intra-ovarian abundance of *Star, Cyp11a1, Cyp17a1, Hsd3b1* and *Ar* transcripts in the ovaries of infantile females determined by quantitative real-time RT-PCR and normalized to the mRNA levels of *Ppid* (Cyclophilin D) (7 to 9 ovaries from different females/group). In graphs, bars are the means ± SEM. Data were analyzed using a one-way ANOVA test (Kruskal-Wallis in **C**, Tukey’s Multi-Comparison test in **D–H**). Distinct letters indicate significant differences between groups.

LH levels are elevated during mini-puberty in the mouse (1, 2, 4), but whether they promote androgen synthesis through their action on their biosynthesis pathways (schematized in **Figure 2A**) is not known. We, thus, sought whether LH could already drive testosterone synthesis and AR expression at this stage. We used a mouse model with pharmacological reduction of gonadotropin levels by the GnRH receptor antagonist Ganirelix, at a stage when LH levels surge between 12 and 14 dpn (4, 26). We supplemented these mice with the exogenous gonadotropin hCG to replace LH (**Figure 2B**). Ganirelix injection leads to a reduction by ~80% and ~95% of circulating FSH and LH levels, respectively (4). It induced a ~85% decrease in intra-ovarian testosterone contents in comparison with control mice injected with saline (**Figure 2C**). The supplementation of Ganirelix-treated mice with hCG restored intra-ovarian testosterone abundance to that of control mice (**Figure 2C**). In line with these findings, the analyses of transcript levels of several components of the androgen biosynthesis pathway, i.e., *Star* (steroid acute regulatory protein), *Cyp11a1*, *Cyp17a1*, and *Hsd3b1*, showed that their relative intra-ovarian levels, except that of *Hsd3b1*, were downregulated in Ganirelix-treated females as compared with those in controls. The relative levels of *Cyp11a1* and *Cyp17a1* returned to those of control ovaries and those of *Star* became higher upon co-treatment with hCG, suggesting that mini-pubertal LH plays an important role in the synthesis of testosterone by regulating the levels of these transcripts (**Figures 2D–G**). The relative abundance of intra-ovarian *Ar* mRNAs showed a ~20% increase in Ganirelix-treated females as compared with that in controls, suggesting that it was repressed by gonadotropins. The co-treatment with hCG restored *Ar* transcript abundance to that of control females (**Figure 2H**). Overall, these data suggest that LH regulates androgen signaling during mini-puberty.

### AR actions on follicle growth and atresia at mini-puberty

**Figure 3:**
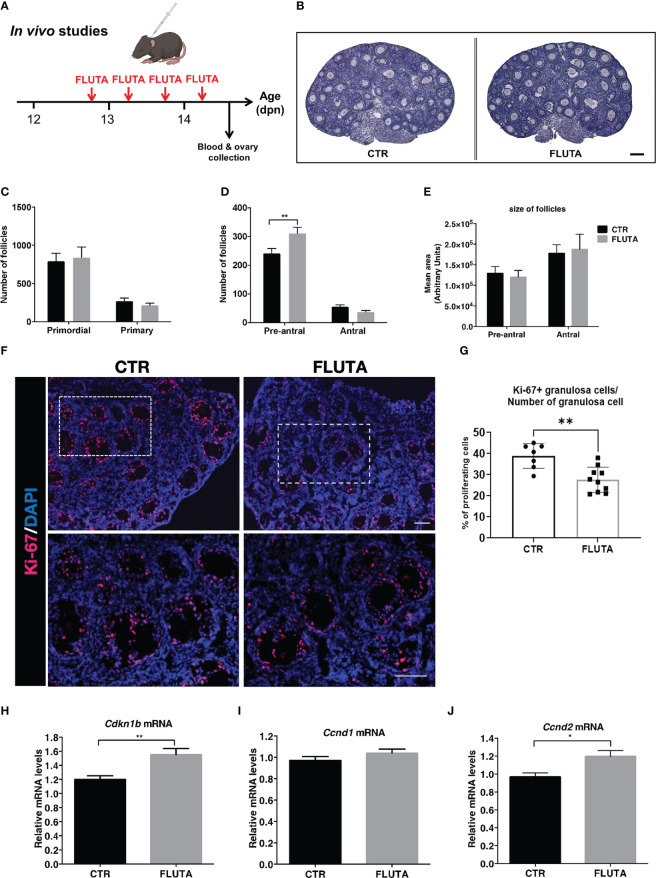
Impact of the inhibition of AR signaling by flutamide on follicular growth in mini-pubertal ovaries **(A)** Schematic representation of the pharmacological procedure used to block AR signaling between 12 and 14 dpn in female mice. **(B)** H/E-stained histological sections of ovaries from a control (CTR) and flutamide (FLUTA)-treated mouse. Bar: 100 µm. **(C, D)** Counts of primordial, primary, preantral and early antral follicles in the ovaries of controls (CTR) and flutamide (FLUTA)-treated mice (n=5 ovaries from 5 females/group). **(E)** Measurement of preantral and early antral follicle areas in the ovaries of controls (CTR) and flutamide (FLUTA)-treated mice (n=5 ovaries from 5 females/group). **(F, G)** *In situ* immunofluorescence analyses of the proliferation cell marker Ki-67 in preantral and antral follicles in controls (CTR) and in flutamide (FLUTA)-treated mice (n=4 to 5 ovaries from 4-5 females/groups). The bottom panels represent higher magnification of the rectangle area delimited in top panels. Ki-67 positive cells exhibit a red fluorescent staining, while cell nuclei appear in blue following DAPI staining. Bars: 100 µm. **(H–J)** Relative intra-ovarian abundance of *Cdkn1b, Ccnd1* and *Ccnd2* transcripts in controls (CTR) and in flutamide (FLUTA)-treated mice, as determined by quantitative real-time RT-PCR and normalization to the mRNA levels of *Ppid* (*Cyclophilin D)* (9 to 11 ovaries from different females/group). In graphs, bars are the means ± SEM. Data were analyzed using a two-way ANOVA test **(C–E)**, a Mann-Whitney test **(G)** and a Student t-test **(H–J)**. *, *P*<0.05; **, *P*<0.01.

We next investigated whether androgens could regulate follicular growth in mini-pubertal ovaries, which contain many preantral and early antral follicles. Because androgens are produced by the ovary during this period, we adopted a pharmacological approach inhibiting their action with the AR antagonist flutamide. We subcutaneously administered flutamide twice daily from 12 to 14 dpn (**Figure 3A**), and we analyzed follicular growth at the end of the treatment by evaluating the number of follicles present at this stage belonging to each stage, i.e., primordial, primary, preantral and early antral follicles, by morphometric studies. Examination of tissue sections revealed no evident gross abnormalities in flutamide-treated mice when compared with controls (**Figure 3B**). The results showed that the number of primordial and primary follicles was not significantly affected by flutamide treatment (**Figure 3C**). However, it significantly increased the number of preantral follicles by about 20% compared to controls, and it had no effect on the number of early antral follicles (**Figure 3D**). Measurement of preantral and antral follicle areas revealed that the size of these two follicular populations did not differ between the two groups (**Figure 3E**), even when the analyses considered the 5 largest follicles of each category (data not shown). The morphological analysis of preantral and early antral follicles did not reveal the presence of atretic follicles (see the criteria in materials and methods).

To determine whether the treatment could affect granulosa cell proliferation in preantral and antral follicles, we performed *in situ* analyses of the proliferating cell marker Ki-67 in the ovaries of control and flutamide-treated females (**Figure 3F**). It was present in granulosa cells of virtually all growing follicles in both groups. However, quantification of the % of Ki-67 positive granulosa cells in preantral and antral follicles indicated that these follicles had a lower proliferation rate in flutamide-treated mice than in control mice (38.9% of Ki-67 positive granulosa cells in controls, *versus* 25.6% in flutamide-treated mice; *P*=0.0046) (**Figure 3G**). As androgens have also been shown to promote or suppress granulosa cell apoptosis depending on studies (27, 28), we sought whether flutamide treatment could have some effects on this process in mini-pubertal ovaries. Consistent with our morphological observations that there is no follicular atresia at this stage, we found no apoptotic cells in control ovaries, as revealed by the absence of cleaved caspase-3 immunodetection (**Supplementary Figure 1**). We observed few apoptotic granulosa cells in preantral and antral follicles of flutamide-treated mice (1-2 apoptotic granulosa cells/ovarian section) (**Supplementary Figure 1**), suggesting that the treatment has very minor effect on granulosa cell apoptosis. As preantral follicles could undergo atresia following an autophagy-related process independent of apoptosis (29), we also evaluated the expression of two autophagy markers, i.e. the protein p62 (the sequestosome 1 abbreviated as SQSTM1) and the autophagy-related gene 7 protein (ATG-7) by immunofluorescence on serial ovarian sections (30). In peripubertal ovaries used as controls, we observed p62/SQSTM1 expression in granulosa cells and oocytes of both preantral and antral follicles (**Supplementary Figure 2**). There was a weak ATG-7 staining in oocytes and thecal cells of growing follicles (**Supplementary Figure 2**). We observed that preantral follicles undergoing follicular atresia, as suggested by their weak AMH and Ki-67 expression, displayed increased expression of ATG-7 in granulosa cells and oocytes and either a decrease or an increase in P62/SQSTM1 expression in granulosa cells.

In mini-pubertal ovaries, p62/SQSTM1 was expressed in granulosa cells and oocytes of growing follicles. ATG-7 was essentially detected in oocytes of growing follicles and it was barely detectable in thecal or granulosa cells. AMH was present in growing follicles located at the periphery of the ovary, and weak or absent from the preantral/early antral stages located toward the center of the ovary, as previously shown in mice and rats at this stage (26, 31). We recently demonstrated that the high FSH levels at mini-puberty mediate the loss of AMH expression and promote the expression of *Cyp19a1* in these follicles, therefore suggesting their FSH receptivity (26). Importantly, we found no difference in the expression of autophagy markers and AMH in preantral and early antral follicles between the control and flutamide-treated groups, despite the marked decrease in the % of Ki-67 positive cells in the flutamide-treated group, as compared with the control group (**Supplementary Figure 2**).

We studied the molecular mechanism underlying AR actions on follicle growth in the mini-pubertal ovary by analyzing the relative expression levels of a factor inhibiting cell cycle progression, i.e., p27Kip1(*Cdkn1b)*, which is an inhibitor of the cyclin dependent kinases (32). The relative abundance of *Cdkn1b* transcripts increased by about 20% in flutamide-treated females compared with controls (**Figure 3H**). We also analyzed the relative expression levels of factors promoting cell proliferation, including Cyclin D1 (*Ccnd1)* and Cyclin D2 (*Ccnd2*), which are required for cell progression through the G1 phase of the cell cycle (32, 33). The relative expression levels of *Ccnd1* were not altered by flutamide treatment (**Figure 3I**), unlike those of *Ccnd2* showing significant up-regulation (**Figure 3J**). *In situ* studies with a specific Cyclin D2 antibody showed that this protein was specifically expressed in granulosa cells of primary and preantral follicles located at the periphery of the ovary and not in preantral and antral follicles located in the center, in controls as in flutamide-treated females (**Supplementary Figure 3**). Taken together, these data suggest that androgens could have some effects on follicular growth by modulating granulosa cell proliferation during mini-puberty.

### AR actions on follicular maturation at mini-puberty

**Figure 4:**
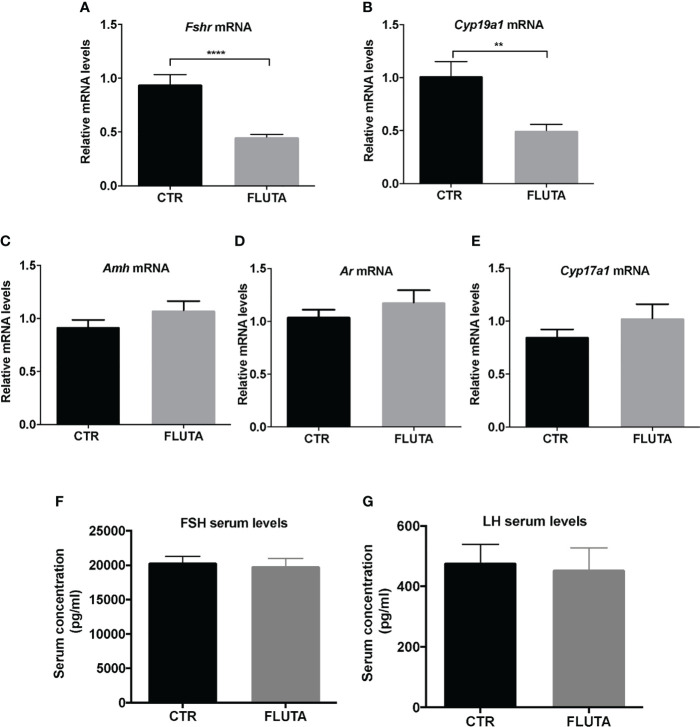
Impact of the inhibition of AR signaling by flutamide on key markers of folliculogenesis and gonadotropin levels **(A–E)** Relative intra-ovarian abundance of *Fshr*, *Cyp19a1*, *Amh*, *Ar*, and *Cyp17a1* transcripts in controls (CTR) and in flutamide (FLUTA)-treated mice. It was determined by quantitative real-time RT-PCR and normalized to the mRNA levels of *Ppid* (Cyclophilin D) (9 to 11 ovaries/group). **(F, G)** Serum FSH and LH levels measured by Luminex technology in controls (CTR) and in flutamide (FLUTA)-treated mice (7 to 10 females/group). In graphs, bars are the means ± SEM. Data were analyzed using a Student t-test. **, *P*<0.01; ****, *P*<0.0001.

We investigated whether AR signaling could contribute to follicular maturation, as reported in the adult ovary (16). We, thus, investigated by RT-qPCR the possible changes induced by flutamide treatment in the relative expression of key genes involved in steroidogenesis and folliculogenesis, i.e., *Cyp19a1*, *Cyp17a1*, *Amh*, *Fshr* and *Ar*. Interestingly, the relative abundance of both *Fshr* and *Cyp19a1* mRNAs decreased by about 2-fold following flutamide treatment compared with controls (**Figures 4A, B**), while that of the other studied transcripts did not significantly change, including that of *Amh*, consistent with our *in situ* studies (**Figures 4C–E**, **Supplementary Figure 2**). To determine whether the alteration in *Cyp19a1* and *Fshr* mRNA levels resulted from a possible change in gonadotropin levels induced by flutamide, we measured LH and FSH serum levels. However, neither FSH nor LH circulating levels were impacted by flutamide treatment during mini-puberty (**Figures 4F, G**), suggesting that the observed alterations in ovarian gene expression rely on direct AR-mediated regulation in the mini-pubertal ovary.

### Determination of the mechanism by which AR signaling regulates Cyp19a1 expression in mini-pubertal ovaries

**Figure 5:**
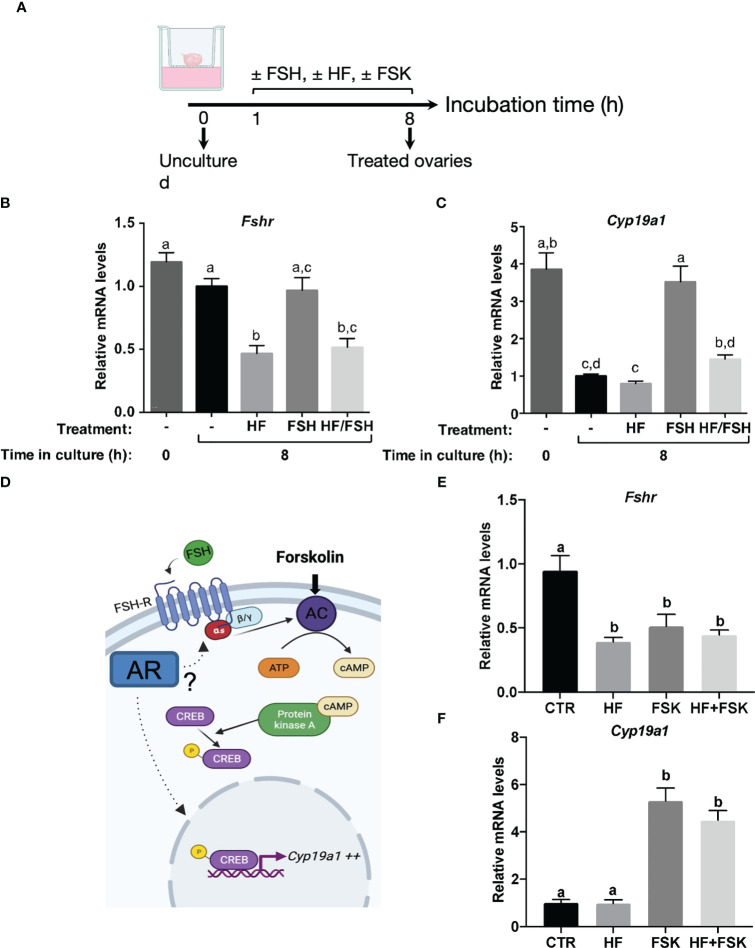
Molecular mechanism whereby AR interferes with FSH-induced expression of *Cyp19a1* **(A)** Schematic representation of the procedure used for ovarian culture. **(B, C)** Relative intra-ovarian abundance of *Fshr* and *Cyp19a1* transcripts in ovaries cultured for 8h with vehicles (-), hydroxyflutamide (HF, 10 µM), FSH (100 ng/ml), hydroxyflutamide (10 µM) plus FSH (100 ng/ml) (HF/FSH), or uncultured (time 0). It was determined by quantitative real-time RT-PCR and normalized to the mRNA levels of *Ppid* (Cyclophilin D) (7 to 18 ovaries from different females/group). **(D)** Schematic representation of the FSH-mediated activation of *Cyp19a1* expression *via* adenylate cyclase (AC) and protein kinase A (PKA). FSH binds to its G protein-coupled receptor FSHR to activate AC, thereby leading to cAMP accumulation and PKA activation. PKA phosphorylates CREB, which is translocated into the nucleus where it induces *Cyp19a1* transcription. **(E, F)** Relative intra-ovarian abundance of *Fshr* and *Cyp19a1* transcripts in organotypic cultures of ovaries treated with vehicles (CTR), hydroxyflutamide (HF), forskolin (FSK) and hydroxyflutamide plus forskolin (HF+FSK). It was determined by quantitative real-time RT-PCR and normalized to the mRNA levels of *Ppid* (Cyclophilin D) (7 to 8 ovaries from different females/group). In graphs, bars are the means ± SEM. Data were analyzed using a one-way ANOVA test. Distinct letters indicate significant differences between groups.

We next investigated whether the AR-mediated regulation of *Cyp19a1* expression observed in this study implies FSH signaling, and in particular the regulation of the abundance of *Fshr*, which is a known AR target in the ovary (17–19). We carried out organotypic cultures with mini-pubertal ovaries to determine the relative expression levels of *Fshr* and *Cyp19a1* in response to treatment with hydroxyflutamide (HF) (used instead of flutamide because it is already active in blocking the AR pathway, unlike flutamide which needs to be metabolized *in vivo*) or with a high FSH concentration mimicking that of mini-puberty (**Figure 5A**) (4). In ovaries cultured for 8 hours under control conditions, *Fshr* mRNA abundance remained stable while that of *Cyp19a1* mRNA dropped sharply compared to uncultured ovaries, indicating that FSH stimulation may be required for maintaining basal levels of *Cyp19a1* mRNA (**Figures 5B, C**). The treatment by HF, either alone or combined with FSH, down-regulated *Fshr* expression by about 50% compared to control ovaries, showing that HF treatment efficiently counteracted the actions of androgens produced by ovarian explants (**Figure 5B**). In contrast, HF had no effect on the relative expression of *Cyp19a1*, which remained at the same low levels seen in untreated control ovaries (**Figure 5C**). However, it significantly suppressed the FSH-induced elevation in *Cyp19a1* abundance, decreased by about 2.5-fold in the group of HF/FSH-treated ovaries compared to the FSH-treated group (**Figure 5C**).

It is generally accepted that FSH up-regulates the expression of the *Cyp19a1* gene upon binding to its receptor leading to activation of several down-stream signaling pathways among which adenylate cyclase/PKA plays a major role (34) (**Figure 5D**). We then sought to determine whether the observed HF-induced down-regulation of *Cyp19a1* in FSH-treated ovaries may result from a decreased activity of FSH signaling resulting from the decrease in *Fshr* transcripts, rather than a direct effect on *Cyp19a1* gene expression. To this end, we used forskolin (FSK) to activate the adenylate cyclase, and under this condition we analyzed the effect of HF on the relative levels of *Fshr* and *Cyp19a1* expression. In agreement with our previous results, HF treatment alone decreased by about 2-fold *Fshr* expression and had no effect on that of *Cyp19a1* (**Figures 5E, F**). The treatment with FSK alone also decreased *Fshr* expression by about 1.5-fold compared with the control group, and it significantly increased that of *Cyp19a1*, as expected (**Figures 5E, F**). Importantly, addition of HF to FSK did not decrease the relative abundance of *Fshr* and *Cyp19a1* transcripts, unlike addition of FSH (**Figures 5B–F**). Taken together, these findings support the idea that the androgens would regulate *Cyp19a1* expression by acting up-stream FSH-mediated activation of adenylate cyclase.

## Discussion

Although androgen-mediated regulation of ovarian function during reproductive life has been demonstrated, there was no information on the possible roles of androgens in the ovary during mini-puberty. By manipulating the AR signaling pathway *in vivo* and in cultured ovaries, we provide evidence for the first time that androgens already play an important role during this developmental stage as a contributor of follicular growth and ovarian endocrine activity.

Our study of androgen production indicates that mouse ovaries already produce testosterone during mini-puberty, although at much smaller amounts than during late juvenile period and reproductive life. Our *in vivo* experiments indicate that this production is highly dependent upon the LH pathway, since hCG treatment efficiently restored testosterone production in Ganirelix-treated females, possibly by stimulating the expression of *Star*, *Cyp11a1* and *Cyp17a1*. Furthermore, we provide evidence that the ovaries are already androgen-responsive as suggested by the observed AR expression in growing follicles from the primary stage onwards. Manipulation of the LH pathway *in vivo* with experiments using Ganirelix-treated mice supplemented or not with hCG showed that *Ar* transcript abundance was repressed by LH signaling. These findings suggest that the elevated levels of mini-pubertal LH play an important role in regulating androgen responsiveness in the ovary at this period of life. These data, along with our previous observation that FSH promotes E2 synthesis by the ovary (4), provide additional clues to support the idea that the higher elevation in FSH and LH circulating levels seen in premature infants can be responsible for high estrogen and androgen levels (8–10).

The possibility that androgens regulate ovarian function during mini-puberty is supported by our studies using pharmacological approaches. Indeed, we observed that the treatment with the AR antagonist significantly increased the number of preantral follicles. We currently do not know whether this surfeit of preantral follicles resulted from increased recruitment of primordial follicles, increased basal follicular growth and/or decreased growth to the antral stage, as we observed no changes in the number of follicles in the other categories. Our morphological studies and immunofluorescence analyses of cleaved caspase-3, p62/SQSTM1 and ATG-7 together with that of AMH and Ki-67 suggest that it did not result from an alteration in follicular atresia, which may still be absent at this stage in rodent ovaries (26, 35) although one cannot exclude that follicular atresia occurred following another form of cell death that we did not study, since additional death pathways have been reported in the ovary (36). One study suggests the occurrence of follicular atresia during mini-puberty in mice, but there is very few information on the methods used to analyze this process and this was analyzed in another strain of mice (Kunming mice) showing distinct ovarian maturation than the strain we used, as suggested by the ontogenesis of follicular markers (26, 37).

Androgens have been reported to stimulate primordial follicle recruitment in mice and monkeys as well as basal follicular growth (22, 28, 38). It could also promote the transition from the preantral to the antral stage by inducing FSH receptivity in preantral follicles (20–22). In our experiments, flutamide treatment robustly decreased the relative abundance of *Fshr* mRNAs, suggesting that it lowered FSH receptivity. It also led to decreased granulosa cell proliferation. Our analysis of the cyclin-dependent kinase inhibitor, p27KIP1, revealed the up-regulation of its transcript, *Cdkn1b*, in flutamide-treated mice. In the mouse ovary, this protein is expressed in granulosa cells, thecal cells and oocytes (39, 40). *Cdkn1b* knock-out mice display aberrant activation of primordial follicles, suggesting that this protein negatively regulates follicle growth (39). Although we presently do not know in which cell type(s) and follicle(s) *Cdkn1b* was up-regulated in flutamide-treated mice, our study of Ki-67 expression leads us to hypothesize that this occurred in granulosa cells of preantral/antral follicles. Our study of D-type cyclin-dependent kinase activators contributing to cell cycle progression revealed that the treatment also up-regulated Cyclin D2 transcript abundance, while it had no effect on that of Cyclin D1. Both cyclins are expressed in the ovary, but Cyclin D1 is present in thecal cells while Cyclin D2 is expressed in granulosa cells of growing follicles (4, 32). Cyclin D2 is involved in granulosa cell proliferation, and its deletion in *Ccnd2^-/-^* mice leads to the arrest of follicle growth at the preantral stage (33). A possible explanation for the apparent contradictory findings on flutamide-induced Cyclin D2 expression despite reduced granulosa cell proliferation could be that the balance between P27KIP1 and Cyclin D2 is in favor of P27KIP1 action. On the other hand, during mini-puberty, Cyclin D2 is expressed in primary and preantral follicles located at the periphery of the ovary and absent in preantral and antral follicles located in the center, suggesting that granulosa cell proliferation is not necessarily mediated by Cyclin D2 in follicles of the first follicular waves, as previously shown (4). The fact that flutamide treatment did not alter the expression pattern of Cyclin D2 within the ovary suggests that the induced upregulation of *Ccnd2* mRNA abundance occurred specifically in primary and preantral follicles of the periphery. This up-regulation of *Ccnd2* expression may also simply reflect the increased number of preantral follicles expressing Cyclin D2. Taken together, these observations suggest that the treatment may have affected the growth of preantral follicles to the antral stage, thereby leading to the accumulation of preantral follicles. Following this interpretation, we propose that androgens may support the growth of follicles from the preantral to the antral stage during mini-puberty, as reported in other studies (19, 22, 41).

Importantly, we found that flutamide treatment markedly down-regulated *Cyp19a1* aromatase expression in mini-pubertal ovaries, while it had no significant alteration on the abundance of *Cyp17a1*, *Amh* or *Ar* transcripts. These findings imply that androgens would regulate follicle maturation by stimulating *Cyp19a1* expression in growing follicles present in the ovary at this stage. This effect of androgens has been described in other studies carried out on cultured mouse preantral follicles or bovine granulosa cells (18, 19). The absence of regulation of *Amh* and *Ar* transcripts seen in our study contrasts with previous observations performed *in vitro* showing that androgens reduce their expression (19, 22). This apparent discrepancy may result from the different experimental parameters between our *in vivo* study considering the whole ovary and these *in vitro* studies restricted to preantral follicles.

It is well documented that FSH signaling is necessary to stimulate *Cyp19a1* expression in granulosa cells of the adult ovary, and that this action of FSH can be modulated by androgens (42–44). We found that flutamide-induced down-regulation of *Cyp19a1 in vivo* occurred concurrently to suppressed *Fshr* expression and without alteration in circulating FSH levels. According to our organotypic culture experiments, the down-regulation of *Cyp19a1* expression by hydroxyflutamide occurred in an FSH-dependent manner, unlike that of *Fshr*. Furthermore, when the action of FSH was replaced by forced PKA pathway activation with forskolin treatment, hydroxyflutamide treatment had no more effect on the FSH-induced *Cyp19a1* expression. Overall, these findings suggest that androgens amplify FSH action on *Cyp19a1* expression during mini-puberty, similar to its action during reproductive life. We presently do not know how androgens regulate *Fshr* and *Cyp19a1* expression during mini-puberty. Recent studies performed in mouse granulosa cells indicate that androgens lower the H3K27me3 marks (a gene silencing epigenetic mark) present in these two genes, thereby up-regulating their expression (45). That would occur independently of direct AR binding to androgen response elements, and the precise mechanism is yet to be discovered (45). From our experiments and those of others, we assume that androgens regulate *Cyp19a1* expression to some extent through their action on *Fshr* expression, as proposed in other mammal species (17–19).

**Figure 6:**
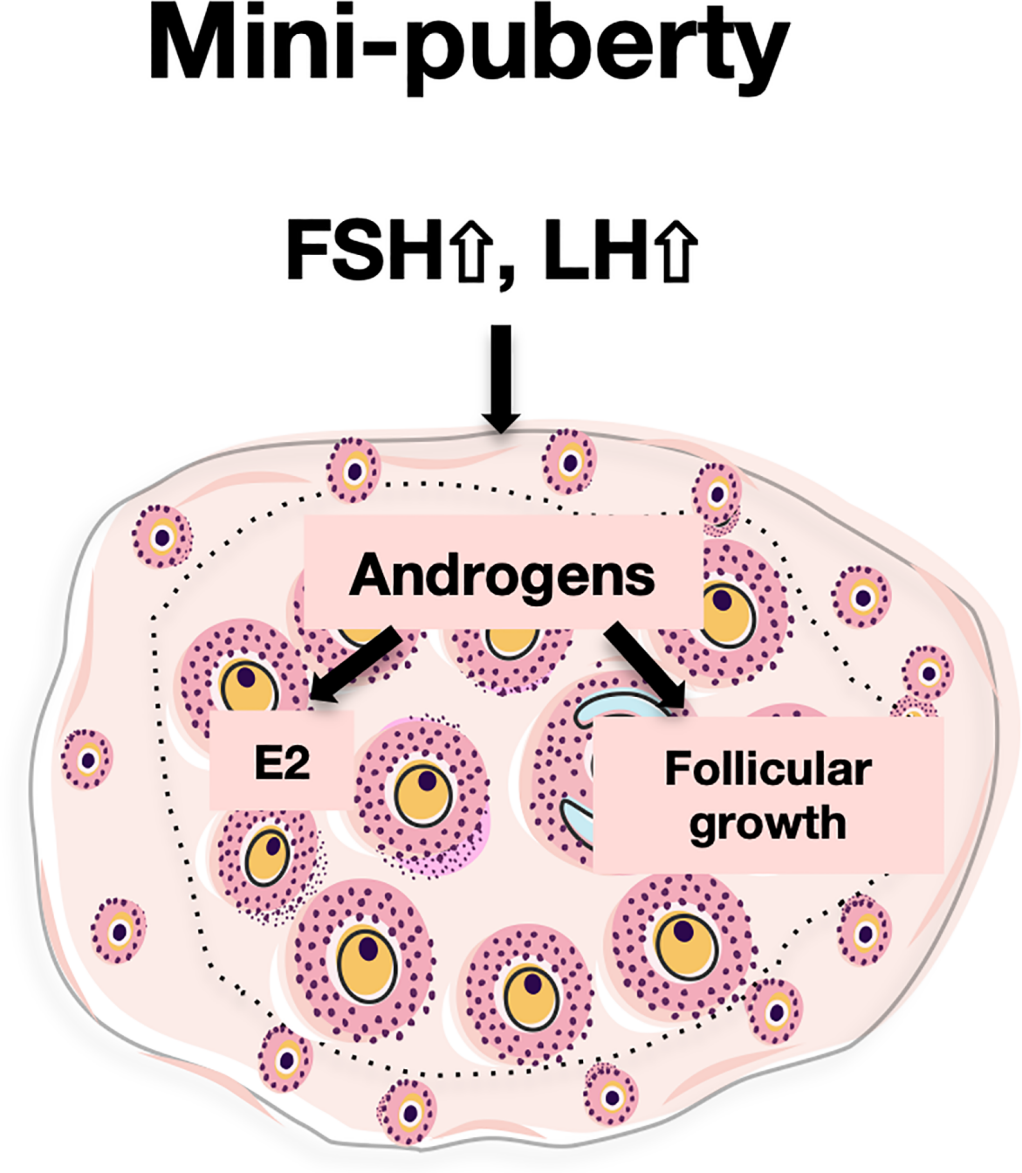
Proposed actions of androgens in the mini-pubertal ovary During mini-puberty, the elevation of LH allows the ovarian synthesis of androgens. Androgens would act through their nuclear receptors (AR) to enhance FSH receptivity in growing follicles. This, together with high FSH levels, would stimulate *Cyp19a1* expression and estradiol (E2) synthesis. In addition, androgens would stimulate the growth of preantral follicles to the antral stage.

Overall, our data suggest that androgens regulate two major processes in the mini-pubertal ovary, i.e., steroidogenesis and follicular growth, in interaction with gonadotropins (**Figure 6**). Indeed, LH appears to regulate androgen synthesis and receptivity in growing follicles. In turn, AR signaling may contribute to FSH stimulation of estradiol synthesis, a process which mainly occurs in the first follicular waves (4). AR signaling may also participate in the growth of these follicles to the antral stage. These rapidly growing follicles are massively eliminated by atresia during the juvenile period in rodents, but some of them are ovulated at puberty and at the very beginning of reproductive life (14, 15). The intra-ovarian action of AR signaling at mini-puberty may, thus, play an important role in the growth state and endocrine behavior of these follicles. Contrasting with their deleterious actions on the ovary leading to PCOS when present at supraphysiological levels during prenatal and early postnatal life (46), we propose that mini-pubertal androgens could play an important physiological role in the ovary, and by extension, implement future reproductive function. Following this interpretation, it may be of interest to investigate whether preterm babies, which may present with higher levels of androgens (10), could display reproductive health disorders.

## Data availability statement

The raw data supporting the conclusions of this article will be made available by the authors, without undue reservation.

## Ethics statement

The animal study was reviewed and approved by Institutional Animal care and Use committee of the University Paris Cité and by the French Ministry of Agriculture (agreement #04015.01).

## Author contributions

MD, CF and CG designed and performed the experiments, and analyzed the data. MC, RC, FG and VC performed experiments. MD, MC and CG prepared the figures and wrote the original manuscript. All authors contributed to the article and approved the submitted version.
