# Supplementary material for: Androgen receptor signaling regulates follicular growth and steroidogenesis in interaction with gonadotropins in the ovary during mini-puberty in mice
Source: Front Endocrinol (Lausanne). 2023 Apr 19;14:1130681. doi: 10.3389/fendo.2023.1130681 (PMC10154677; doi:10.3389/fendo.2023.1130681)
Supplement: Supplementary file 1 [file DataSheet_1.pdf]

Supplementary Figure 1

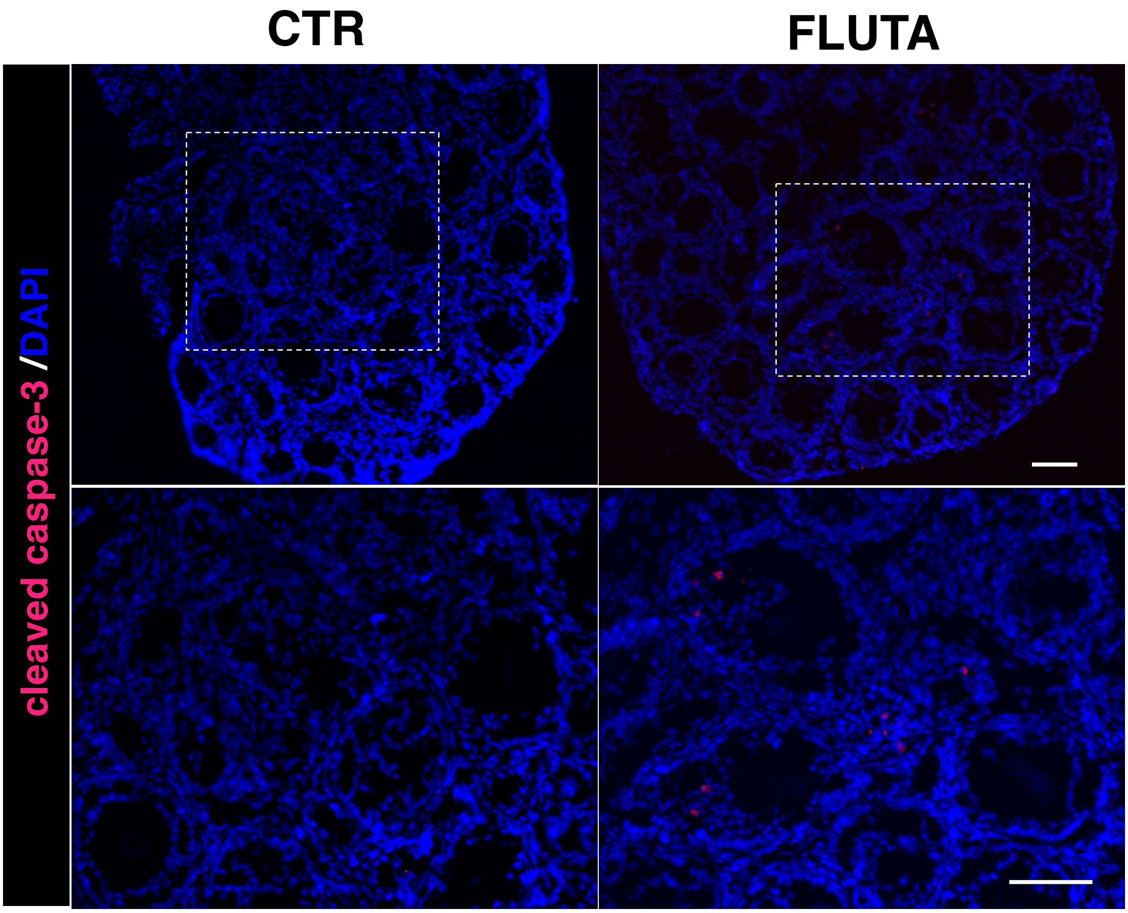

In situ immunofluorescence analyses of the apoptosis cell marker cleaved caspase-3 in preantral and antral follicles in controls (CTR) and in flutamide (FLUTA)-treated mice (n=4 to 5 ovaries from 4-5 females/groups). Cleaved caspase-3 positive cells exhibit a red fluorescent staining, while cell nuclei appear in blue following DAPI staining. Bars: 100 µm.

Supplementary Figure 2

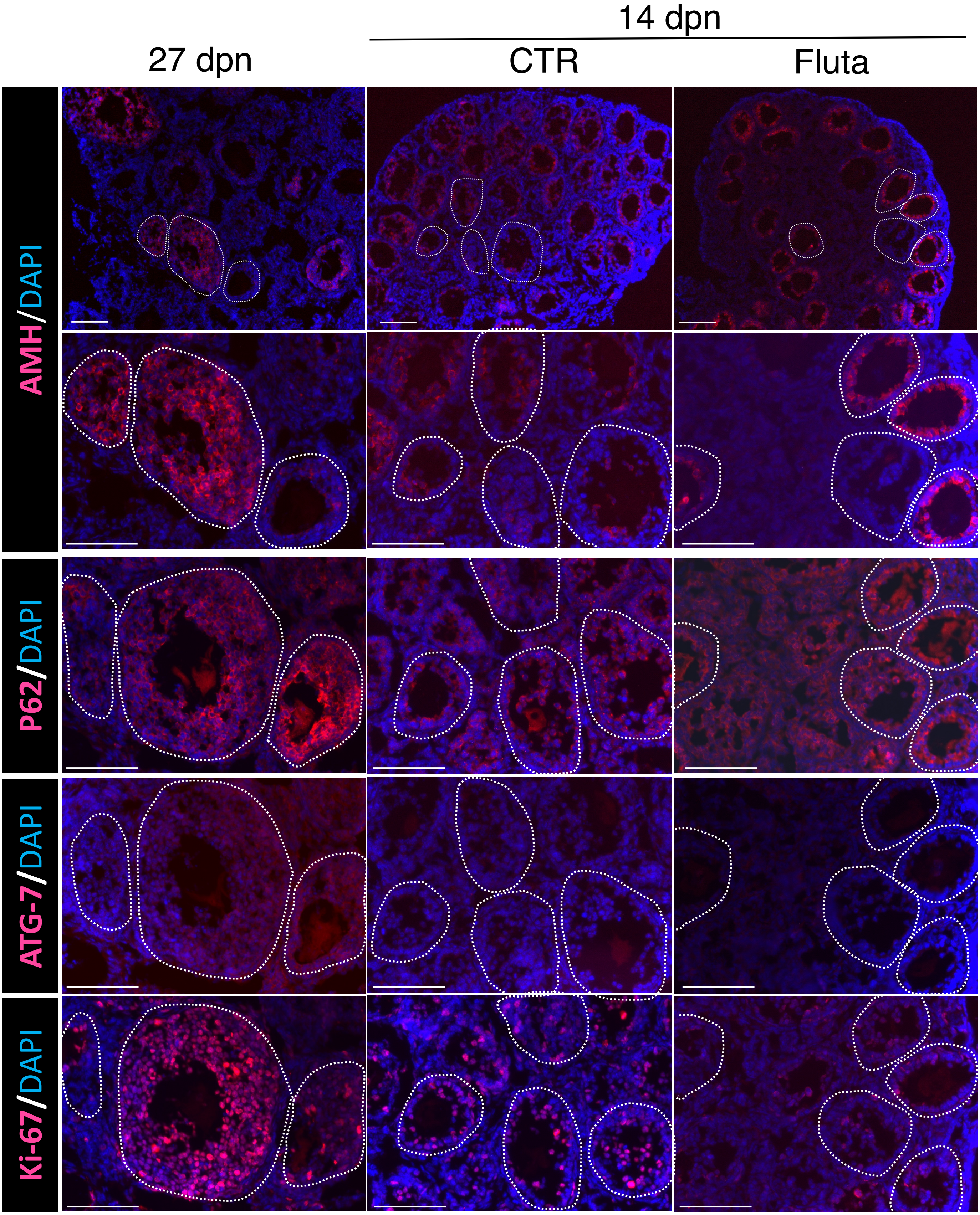

In situ immunofluorescence analyses on serial ovarian sections of AMH, autophagy cell markers p62/STQSM1 and ATG-7, and Ki-67 in preantral and antral follicles in the ovaries of a peripubertal control female and of 14 dpn controls (CTR) and flutamide (FLUTA)-treated mice (n=4 to 5 ovaries from 4-5 females/groups). Pictures show merged images of red immunofluorescent and DAPI staining. The top panels show merged images of AMH and DAPI. Outlined follicles appear in higher magnifications in the other panels. Preantral and antral follicles are delimited by dotted lines. Bars: 100 µm.

Supplementary Figure 3

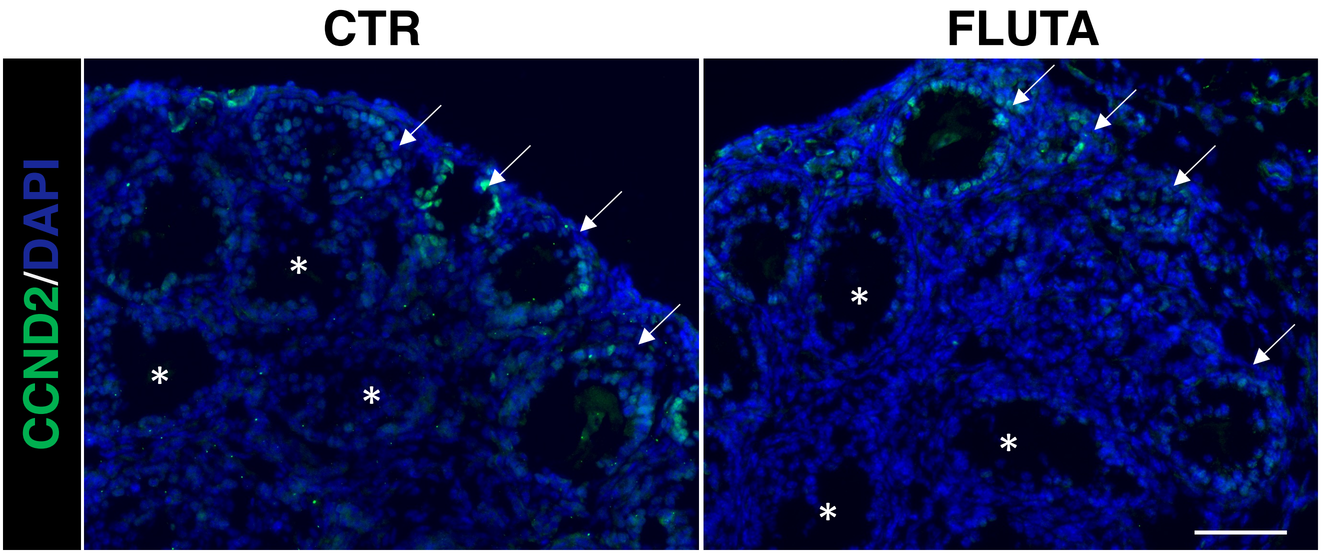

Immunofluorescence detection of cyclin D2 (CCND2) in the ovaries of control (CTR) and flutamide (FLUTA)-treated mice. Only primary and preantral follicles located at the periphery express CCND2 (arrows). Preantral/early antrall follicles located toward the center are negative (star). Bar: 100 µm.
